# Variability and symmetry of gait kinematics under dual-task performance of older patients with depression

**DOI:** 10.1007/s40520-022-02295-6

**Published:** 2022-11-18

**Authors:** Pia Jungen, João P. Batista, Miriam Kirchner, Ute Habel, L. Cornelius Bollheimer, Charlotte Huppertz

**Affiliations:** 1 Department of Psychiatry, Psychotherapy and Psychosomatics, Faculty of Medicine, RWTH Aachen University, Pauwelsstraße 30, 52074 Aachen, Germany; 2 Department of Geriatrics, Faculty of Medicine, RWTH Aachen University, Morillenhang 27, 52074 Aachen, Germany; 3 School of Physical Therapy, Campus Rheinland, SRH University of Applied Health Sciences, 51377 Leverkusen, Germany; 4 Alexianer Aachen GmbH, Alexianergraben 33, 52062 Aachen, Germany; 5 Institute of Neuroscience and Medicine 10, Research Centre Jülich, Wilhelm-Johnen-Straße, 52428 Jülich, Germany

**Keywords:** Fall risk, Old age, Three-dimensional gait analysis, Spatiotemporal, Toe clearance, Dual-task

## Abstract

**Background:**

Depression in old age is associated with an increased fall risk. Especially in cognitively challenging situations, fall-promoting gait deviations could appear due to depression- and age-related cognitive deficits.

**Aim:**

This study investigates (i) whether there are differences in gait performance between depressed older patients and healthy controls and (ii) if gait patterns aggravate when performing a cognitive task whilst walking.

**Methods:**

16 depressed older patients (mean age: 73.1 ± 5.8 years) and 19 healthy controls (mean age: 73.3 ± 6.1 years) were included in the study. Spatiotemporal gait parameters (speed, stride length, swing time) and minimum toe clearance were recorded using a three-dimensional motion-capture system under a single- and a dual-task condition (counting backwards).

**Results:**

After Bonferroni correction, depressed older patients showed significantly slower walking speed, shorter strides and smaller minimum toe clearance, as well as greater variability in stride length than healthy controls. Under the dual-task, gait performance deteriorated compared with single-task, with slower gait speed, shorter strides, and longer swing time.

**Discussion:**

Slower walking speed and shorter steps of depressed patients may be a strategy to counteract their fall risk. Increased variability suggests a less stable gait pattern in patients, which could be a reason for their increased fall risk.

**Conclusions:**

Depression in old age has a strong effect on gait performance. Possible interventions that might prevent falls in this vulnerable group are discussed. The study was registered at Open Science Framework on May 18, 2021 (publicly accessible May 30, 2023).

**Supplementary Information:**

The online version contains supplementary material available at 10.1007/s40520-022-02295-6.

## Introduction

Previous studies have shown that depressed older patients in general walk slower compared to healthy peers [1, 2]. This raises the question of farther-reaching deviations in terms of spatiotemporal gait parameters (describing the spatial and temporal placement of the feet) which may cause an unstable gait in depression and, thus, an increased fall risk. So far, only a few studies have addressed the association between spatiotemporal measures and depressive symptoms in older people [2–6]. As part of the Einstein Aging study [2], depressive symptoms were assessed in 610 community-residing adults (39% male, mean age: 80.3 years) without dementia and gait was recorded using a computerized walkway with integrated pressure sensors (GAITRite, CIR Systems Inc., Franklin, New Jersey; USA). Velocity, stride length (distance between heel strike of two successive steps of the same foot), and swing time variability (variability of the time that the foot does not touch the ground) were negatively associated with depressive symptoms. In contrast, Paleacu et al. [5] did not find an association between (pre-treatment) depressive symptoms and gait.

Cognitive deficits are a typical symptom of depression [4, 7]. Gait deviations might, thus, be especially prominent in cognitively demanding situations, such as monitoring the traffic on a busy road whilst walking. A few studies investigated spatiotemporal gait parameters in depressed older patients as they simultaneously conducted a cognitive task [3, 4, 6]. As part of the Irish Longitudinal Study on Aging [3], gait of 1998 community-residing adults (45% male, mean age: 68.2 years) was assessed. Participants were asked to walk in their usual pace without and with an additional task (reciting alternating letters). In addition, late-onset depressive symptoms and the use of antidepressant medication were assessed. The latter was associated with reduced pace and reduced stride length in both conditions, while there was no association between depressive symptoms and any of the gait parameters. In a different study [6], 28 geriatric patients (25% male, mean age: 78.4 years) were asked to count backwards whilst walking. Depressive symptoms were associated with a decreased step length and increased double support time (time in gait cycle when both feet touch the ground), but there was no association with cognitive task. A further study [4] did not find differences between spatiotemporal gait parameters of 15 depressed older patients (mean age: 70.7 years, 40% male) compared to 17 controls (mean age: 75.1 years, 53% male). However, the patient group showed increased variability in stride time, stride velocity, and swing time under dual-task (DT) (reciting alternating letters or counting backwards by 2 s) compared with single-task (ST).

Apart from changes in spatiotemporal gait parameters, reduced minimum toe clearance (MTC) and increased variability in MTC might increase the risk for tripping and falling in older people [8, 9]. MTC describes the minimum vertical distance between toe and surface during the swing phase of a gait cycle. As the distance between toes and the ground is the smallest in the whole swing phase and the foot reaches maximum horizontal velocity, tripping is most probable [10]. Additionally, stability is limited as the body is supported by only one leg in this phase [10] and the body's center of mass shifts in the direction of progression and away from the base of support [11]. Studies that examined MTC in healthy older people tend to find lower MTC and higher MTC variance with higher age [8, 9], an additional cognitive task did not seem to affect MTC in one study [12]. Studies investigating MTC in depressed older patients are lacking.

The present study explores (i) whether there are differences in gait performance between depressed older patients and healthy controls and (ii) the effect on gait of performing a cognitive task whilst walking. The gold standard of gait analysis was used to measure gait with high precision and accuracy on three planes (three-dimensional/3D gait analysis). We focus on those parameters that were most strongly associated with falls in previous studies [8, 9, 13–17]: three spatiotemporal parameters (speed, stride length, swing time) and MTC. In addition to mean values, we were interested in the variability between steps and the symmetry between left and right leg. Both are likely to play a major role in gait stability but are rarely considered [4, 9]. Based on findings of previous studies, we aim to confirm that (i) depressed older patients walk slower, with decreased stride length and increased swing time. Based on studies in healthy older people, we hypothesize that depressed patients would show decreased MTC. We also hypothesize that (ii) depressed patients will show increased variability and decreased symmetry for stride length, swing time and MTC, and that (iii) gait worsens under the DT condition for both participant groups. It is well known that depression in older people is associated with an increased fall risk [7, 18]. Apart from harmful consequences of falling in this age group [7, 17], the fear to fall often leads to the avoidance of physical activity [17]—with the consequence of a deterioration of life quality and both physical and mental health. In the long term, a better understanding of gait deviations might lead to targeted interventions to decrease the fall risk and associated fears in depressed older people.

## Methods

### Participants

Sixteen depressed patients (DP; 4 males) and nineteen healthy controls (HC; 8 males) aged 64 years or older participated in the study. DP were diagnosed by their physician with moderate to severe unipolar depression without psychosis and were recruited from psychiatric wards (*N* = 7), via practice-based psychiatrists and psychotherapists (*N* = 2) and flyers in public spaces (*N* = 7). HC were recruited via flyers in public spaces (*N* = 2), via a university study program (*N* = 11) or university sports for senior students (*N* = 6) at the study location.

All participants were able to understand simple instructions and safely walk 10 m at once. Comorbidities affecting gait (e.g., orthopedic or neurological disorders), mental disorders relevant to the study (e.g., psychosis, dementia), use of highly potent neuroleptics, severe visual impairment, severe obesity (body mass index/BMI > 39), and walking only with pain or with a walking aid led to study exclusion. Written informed consent was obtained from all participants. The study was approved by the local ethics committee (EK058/18), registered at Open Science Framework (publicly accessible from May 30, 2023) and all procedures were in accordance with the Declaration of Helsinki.

### Procedure

Data were collected on two days. On a first visit, questionnaire data were collected on demographics and medication, amongst others. Depression and its severity were quantified based on the Geriatric Depression Scale-Short Form (GDS-SF) [19], the Montgomery-Asberg Depression Rating Scale (MADRS) [20], and the Test for early Diagnosis of Dementia with Differentiation from Depression (TFDD) [21]. Cognitive functioning was assessed with the Mini-Mental State Examination (MMSE) [22] and four cognitive tests, which intended to cover a broad range of cognitive domains: Trail Making Tests (TMT) A and B [23], the Go/No-go test [24], the n-back test [25], and the Stroop test [26]. The Falls Efficacy Scale-International Version (FES-I) [27] assessed fear of falling, the Timed Up and Go test (TUG) was used to rate mobility [28].

Gait analysis was performed on a second day. Participants wore short, sleeveless, and tight-fitting clothes, and non-slip socks. The marker-set protocol adopted for this study comprised 40 retroreflective markers, positioned at anatomically defined bone points, adapted from the lower body CAST marker set [29]. Single markers were placed directly on the skin, while marker clusters were positioned with straps around the segments. Ten infrared cameras (Qualisys AB, 5+ series, Gothenburg, Sweden), combined with the motion-capture system Qualisys Track Manager “QTM” (Qualisys AB, Gothenburg, Sweden), collected biomechanical data with a sampling frequency of 120 Hz.

For an anatomical calibration trial, participants were asked to remain stationary, abduct their arms at a 45-degree angle from the body, and bend their arms at a 90-degree angle. Static markers were then removed from the pelvis, the right and the left segments. To familiarize with the situation, each participant was asked to walk around in the motion laboratory. Data collection consisted of two conditions. Participants were asked to walk 10 m in their usual pace, first without additional task (ST) and secondly with simultaneous performance of a cognitive task (DT), for 10 trials each. The cognitive task consisted of counting backwards in single digits from a three-digit number (range 125–250), randomly generated for each trial.

### Data analysis

Gait data were labeled and exported using QTM Software. The first 2.5 m of each trial were removed to calculate the parameters during steady state walking speed. Only those trials that contained a complete gait cycle were considered for the analyses. To define initial contact (heel strike) and toe off, event boundaries were set on the X- and Z-component of the velocity vectors of heel and second metatarsal phalange marker [30]. To perform the kinematics calculations, Visual 3D software version 2020.05.1 (C-motion Inc., Germantown, MD USA) was used. Raw markers-trajectory data were low-pass filtered by 5 Hz using a 4th-order Butterworth filter.

The following parameters were calculated and examined in the study: (a) speed (mean), (b) stride length (mean, coefficient of variation and symmetry index), (c) swing time (mean, coefficient of variation and symmetry index), and (d) MTC (mean, coefficient of variation and symmetry index). The mean value of speed was calculated by dividing stride length by stride time (time period between consecutive heel contacts of the same foot). The remaining mean values were calculated using the sum of all (valid) steps taken, separately for the left and right foot. Subsequently, the mean values of the right and left foot were merged. The coefficients of variation “CoV” [%] = (standard deviation/mean)*100 were calculated for stride length, swing time and MTC, indicating the variability between individual steps. Symmetry indices (SI) were calculated for these three parameters as the deviation of one lower limb (e.g., left, L) compared with the other (right, R) in percent (zero meaning “no difference”): “SI [%] =|((L–R)/(0.5*(L + R))) *100|”.

The main statistical analyses were conducted using SPSS version 27 (IBM SPSS^®^, Armonk, NY, USA). Due to positively skewed distributions, SI values were square root transformed. Mixed-design ANOVAs (within-subject factor: “ST versus DT”, between-subject factor: “DP versus HC”) were performed. As we tested 10 dependent variables in total and we were mainly interested in main effects (within and between subjects), we assumed a Bonferroni-corrected *α*-level of 0.05/(10*2) = 0.0025.

## Results

Sample characteristics are presented in Table 1. As DP were significantly smaller than HC (159 cm versus 172 cm), the mean values of gait parameters were standardized based on leg length, according to Hof et al. [31]. The leg length of each participant was defined as the mean value of the left and right distance between the hip marker and the ground. Results of both unstandardized and standardized data will be presented.

**Table 1:** Sample characteristics of depressed patients (DP) and healthy controls (HC)

Variable	DP (*N* = 16)	HC (*N* = 19)	*p* value
	Mean ± SD	Mean ± SD	
Age (y)	73.1 ± 5.8	73.3 ± 6.1	0.926
Gender (N)	f = 12; m = 4	f = 11; m = 8	0.295
Height (cm)	159 ± 7.9	172 ± 9.5	0.000*
Weight (kg)	70.4 ± 11.5	78.2 ± 14.6	0.095
BMI (kg/m^2^)	27.7 ± 4.4	26.5 ± 4.5	0.449
Depression			
GDS (points)	4.8 ± 3.9	1.3 ± 1.2	0.002*
MADRS (points)	14.9 ± 5.8	1.2 ± 1.6	0.000*
TFDD 1 (points)	40.8 ± 5.4	44.3 ± 3.7	0.050
TFDD 2 (points)	10.9 ± 4.3	0.4 ± 1.1	0.000*
Cognition			
MMSE (points)	27.6 ± 1.8	28.4 ± 1.5	0.186
TMT A (s)	53.7 ± 23.4	38.9 ± 14.0	0.028*
TMT B (s)	115.3 ± 56.9	75.2 ± 26.6	0.017*
Go/no-go test reaction time (ms)	457.3 ± 73.4	467.9 ± 82.1	0.759
Go/no-go test number of errors	2.4 ± 3.1	1.0 ± 1.3	0.365
n-back test reaction time (ms)	477.3 ± 110.4	451.4 ± 43.2	0.381
n-back test number of misses	2.1 ± 1.6	0.7 ± 0.6	0.009*
Stroop test RCW (s)	45.8 ± 13.5	34.9 ± 5.0	0.007*
Stroop test NCB (s)	60.9 ± 14.4	48.5 ± 7.6	0.011*
Stroop test INT (s)	108.8 ± 44.4	89.0 ± 16.5	0.260
Stroop test INT number of uncorrected errors	0.7 ± 1.0	0.5 ± 0.8	0.711
Stroop test INT number of corrected errors	1.7 ± 2.0	1.2 ± 0.8	0.815
Fear of falling			
FES-I (points)	25.4 ± 9.5	19.2 ± 2.8	0.003*
Mobility			
TUG (s)	10.8 ± 2.4	9.6 ± 1.3	0.353

*p* values for normally distributed data: unpaired *t* tests, *p* values for non-normally distributed data: Mann–Whitney *U* tests, *α < *0.05*

*SD* standard deviation, *BMI* body mass index, *GDS* geriatric depression scale, *MADRS* Montgomery–Asberg depression rating scale, *TFDD 1* test for the early detection of dementia with depression differentiation—part on dementia, *TFDD 2* test for the early detection of dementia with depression differentiation—part on depression, *MMSE* mini-mental state examination, *TMT A* trail making test A (connecting numbers in ascending order), *TMT B* trail making test B (alternately connecting numbers and letters in ascending order), *RCW* read color words, *NCB* name color bars, *INT* interference, *FES-I* falls efficacy scale—international version, *TUG* timed up and go test

DP consistently scored higher on the depression scales than HC and none of the HC reached the cut-off to “depression” on any of the questionnaires. Two DP were not identified as being depressed by any of the questionnaires: according to the GDS, three individuals were mildly depressed, one was moderately depressed, two were severely depressed, and ten individuals were not depressed. According to the MADRS, five were moderately depressed, nine were mildly depressed, and two were not depressed. According to the TFDD 2, eleven were depressed and five were not depressed. Half of the DP and none of the HC were taking antidepressants. MMSE-scores were comparable in both groups, with no cognitive restrictions (all ≥ 24). Overall, DP tended to perform slower and to make more errors on the cognitive tests than HC. DP rated their fear of falling as low (*N* = 2), moderate (*N* = 11) and high (*N* = 3)—for HC, these were 12, 7 and 0. Half of the DP and 37% (*N* = 7) of the HC showed slight mobility impairments, the remaining individuals showed no mobility impairments.

Table 2 depicts the means and standard deviations of the gait parameters, stratified by group and condition. Tables 3, 4, and 5 depict the results of the ANOVAs for unstandardized mean values (see Table 3), CoVs (see Table 4) and SIs (see Table 5). For the unstandardized mean values, a strong main effect of group was present for speed [*F*(1, 33) = 18.48, *p* = 0.000, *ƞ*_p_^2^ = 0.36], stride length [*F*(1, 33) = 25.55, *p* = 0.000, *ƞ*_p_^2^ = 0.44] and MTC [*F*(1, 33) = 12.48, *p* = 0.001, *ƞ*_p_^2^ = 0.27]. DP had slower speed, shorter stride length, and smaller MTC compared to controls. The group effect on mean MTC was no longer significant after standardization based on leg length [*F*(1, 33) = 5.45, *p* = 0.026, *ƞ*_p_^2^ = 0.14] (Online Resource 1). There were significant and strong main effects of condition on mean speed [*F*(1, 33) = 29.63, *p* = 0.000, *ƞ*_p_^2^ = 0.47], mean stride length [*F*(1, 33) = 22.12, *p* = 0.000, *ƞ*_p_^2^ = 0.40] and mean swing time [*F* (1, 33) = 13.75, *p* = 0.001, *ƞ*_p_^2^ = 0.29]. Under DT, participants showed slower speed, shorter stride length and longer swing time compared to ST. For the CoVs, there was only one significant and strong main effect of group on stride length [*F*(1, 33) = 18.61, *p* = 0.000, *ƞ*_p_^2^ = 0.36], with more variability in DP. For the SIs, no significant effects were found. There were no significant interaction effects at all.

**Table 2:** Descriptive of gait parameters for depressed patients (DP) and healthy controls (HC)

	DP (*N* = 16)		HC (*N* = 19)	
	ST	DT	ST	DT
Variable				
Speed (m/s)				
Mean ± SD	1.02 ± 0.21	0.91 ± 0.26	1.29 ± 0.16	1.22 ± 0.20
Stride length (m)				
Mean ± SD	1.05 ± 0.15	1.00 ± 0.16	1.30 ± 0.13	1.27 ± 0.17
CoV (%) ± SD	3.69 ± 1.57	3.97 ± 1.31	2.53 ± 0.67	2.58 ± 0.78
SI (%) ± SD	0.42 ± 0.34	0.39 ± 0.20	0.37 ± 0.28	0.33 ± 0.36
Swing time (s)				
Mean ± SD	0.35 ± 0.03	0.36 ± 0.04	0.35 ± 0.02	0.36 ± 0.02
CoV (%) ± SD	4.09 ± 1.62	4.86 ± 2.16	2.95 ± 0.71	3.24 ± 1.08
SI (%) ± SD	2.09 ± 1.51	3.03 ± 2.08	2.18 ± 1.30	2.20 ± 1.56
Minimum toe clearance (cm)				
Mean ± SD	4.48 ± 0.49	4.33 ± 0.61	4.97 ± 0.34	4.89 ± 0.35
CoV (%) ± SD	8.23 ± 2.54	9.83 ± 5.10	6.59 ± 1.54	6.61 ± 1.69
SI (%) ± SD	5.90 ± 4.08	5.57 ± 6.46	5.68 ± 3.96	5.53 ± 4.83

*ST* single-task, *DT* dual-task, *SD* standard deviation, *CoV* coefficient of variation, *SI* symmetry index, *SI* values based on untransformed data (in contrast to Table 5)

**Table 3:** Results of mixed-design ANOVAs for mean values of gait parameters, NOT standardized based on leg length

	Speed mean				Stride length mean				Swing time mean				Minimum toe clearance mean			
	d*f*	*F*	*p*	*ƞ*_p_^2^	d*f*	*F*	*p*	*ƞ*_p_^2^	d*f*	*F*	*p*	*ƞ*_p_^2^	d*f*	*F*	*p*	*ƞ*_p_^2^
Between-subject effects																
Group	1	18.48	0.000*	0.36	1	25.55	0.000*	0.44	1	0.37	0.545	0.01	1	12.48	0.001*	0.27
Error (group)	33				33				33				33			
Within-subject effects																
Condition	1	29.63	0.000*	0.47	1	22.12	0.000*	0.40	1	13.75	0.001*	0.29	1	8.60	0.006	0.21
Condition*group	1	1.40	0.245	0.04	1	1.73	0.198	0.05	1	1.65	0.208	0.05	1	0.92	0.344	0.03
Error (condition)	33				33				33				33			

*Statistical significance with *α* < 0.0025

*ANOVA* analysis of variance, *df* degrees of freedom, *F F* value, *p* p value, *ƞ*_*p*_^*2*^ partial eta-squared

**Table 4:** Results of mixed-design ANOVAs for coefficients of variation (CoV) of gait parameters

	Stride length CoV				Swing time CoV				Minimum toe clearance CoV			
	d*f*	*F*	*p*	*ƞ*_p_^2^	d*f*	*F*	*p*	*ƞ*_p_^2^	d*f*	*F*	*p*	*ƞ*_p_^2^
Between-subject effects												
Group	1	18.61	0.000*	0.36	1	9.95	0.003	0.23	1	9.33	0.004	0.22
Error (group)	33				33				33			
Within-subject effects												
Condition	1	0.48	0.493	0.01	1	5.37	0.027	0.14	1	1.73	0.198	0.05
Condition*group	1	0.25	0.619	0.01	1	1.11	0.299	0.03	1	1.63	0.210	0.05
Error (condition)	33				33				33			

*ANOVA* analysis of variance, *df* degrees of freedom, *F F* value, *p* p value, *ƞ*_*p*_^*2*^ partial eta-squared, *statistical significance with *α* < 0.0025

**Table 5:** Results of mixed-design ANOVAs for (square root transformed) symmetry indices (SI) of gait parameters

	Stride length SI				Swing time SI				Minimum toe clearance SI			
	d*f*	*F*	*p*	*ƞ*_p_^2^	d*f*	*F*	*p*	*ƞ*_p_^2^	d*f*	*F*	*p*	*ƞ*_p_^2^
Between-subject effects												
Group	1	0.59	0.449	0.02	1	0.41	0.526	0.01	1	0.01	0.927	0.00
Error (group)	33				33				33			
Within-subject effects												
Condition	1	0.16	0.688	0.01	1	1.78	0.191	0.05	1	1.08	0.306	0.03
Condition*group	1	0.56	0.459	0.02	1	2.05	0.162	0.06	1	0.04	0.847	0.00
Error (condition)	33				33				33			

*ANOVA* analysis of variance, *df* degrees of freedom, *F F* value, *p* p value, *ƞ*_*p*_^*2*^ partial eta-squared

## Discussion

The aims of this study were to investigate (i) whether there are differences in mean values, variability and symmetry of kinematic gait parameters between older DP and HC and (ii) a potentially aggravating effect on gait patterns of performing a cognitive task whilst walking. DP showed significantly slower walking speed, shorter strides, and smaller MTC, as well as greater stride length variability than HC. Gait performance deteriorated under DT task, with slower gait speed, shorter strides, and longer swing time.

Our finding that DP walked slower and with shorter strides is consistent with our hypothesis and with results of previous studies [1, 2]. As these two parameters are strongly associated (in our sample, Pearson’s correlations were around 0.9), they will be discussed together. Older people with depression have been shown to be more frail compared with healthy peers [7]. Typical characteristics of frailty are reduced muscle strength, worsened balance, and reduced fitness [7]. These might be a consequence of fatigue, psychomotor delay, and withdrawal from physical activity that typically go along with the disease [7]. In our sample, DP revealed more concerns about falling (FES-I questionnaire) and more mobility impairments (TUG test) than HC. DP might—deliberately and/or automatically—lower their speed (and as a consequence shorten their strides) and/or shorten their strides (and, thus, lower their speed) to counteract their fall risk [17]. In contrast to our hypothesis—but in accordance with Gabel et al. [4]—we found no significant difference in mean swing time between DP and HC. We expected a higher swing time to be a cause for falling in DP, since swing time is characterized by a single leg stand: the longer a person stands on one leg, the more difficult it becomes to maintain stability and balance. DP show a somewhat (non-significantly) larger increase in mean swing time under DT than HC though (Online Resource 2a).

Reduced MTC in DP suggests that they are more vulnerable to unexpected ground contact during walking and therefore more prone to stumble and fall than HC. Although the difference was no longer statistically significant after standardization based on leg length, it might still be of clinical relevance. After standardization, means of DP are still smaller and their SDs larger than those of HC (4.92 cm ± 0.52 versus 5.24 cm ± 0.38 for ST and 4.75 cm ± 0.61 versus 5.16 cm ± 0.39 for DT)—the differences might reach statistical significance in larger samples. Depression is associated with reduced muscle strength [32] that may hamper depressed older patients to lift their foot sufficiently during walking. A previous study [33] examining 36 older people with Parkinson’s disease and 38 controls found that in both groups, shorter stride length was related to lower MTC, suggesting that therapeutic interventions to increase strides might lower fall risk in DP as well.

Although for the variability between steps, only the group difference in stride length reached statistical significance after Bonferroni correction (with a large effect size of *ƞ*_p_^2^ = 0.36), group differences in swing time and MTC pointed in the expected directions, with more variability in DP (Online Resource 2b) and would have reached statistical significance without a correction (*p* = 0.003 and 0.004). The inability to maintain a steady gait rhythm, i.e., showing major changes from stride to stride, leads to an unstable gait, less balance, and thus to a greater fall risk [15]. An improvement in gait rhythm after treadmill training was shown in 36 Parkinson’s disease patients [34]—the treadmill might function as a pacemaker and be a promising tool to reduce gait variability and fall risk in DP as well.

In contrast to our hypothesis, there were no significant differences between groups or conditions regarding the SI values (Online Resource 2c). To date, there are no other studies that have investigated gait symmetry in DP—but there are studies that have examined gait symmetry in healthy older individuals. One study [10] in 126 older people found that good mental health was associated with better gait symmetry. Previous studies have also shown that maintaining gait symmetry requires cognitive resources when walking is impaired and less automatic [35]. Since DP experience bad mental health and have limited cognitive capacity [7], as partly revealed in our data, we expected this to be reflected in greater gait asymmetry. As effects might be too small to be detected with our sample size, future studies should investigate SIs of depressed older patients in large samples.

Differences between ST and DT conditions were significant only for speed, stride length, and swing time (mean values). A higher demand on limited cognitive resources might—either consciously and/or unconsciously—be compensated by slower speed, shorter strides and slower completion of a step (prolonged swing time) to prevent stumbling and falling. It might be helpful to teach patients sensitive attention allocation in this regard and—more generally—to educate them about fall risk in depression and the possible association with cognition. It should be noted that our participants were rather highly educated, with 6/16 (DP) and 11/19 (HC) having the highest possible school leaving certificate in Germany (Abitur), of which 5/16 (DP) and 9/19 (HC) even had an (applied) university degree. Only one DP had no degree. This suggests an originally high level of cognitive performance that might compensate for (some of the) cognitive impairments due to age and/or disease. Additionally, 9 of 16 DP were not in acute inpatient treatment at study time; it is possible that the effect would have been stronger if only in-patients with severe depression had been included.

Intake of certain drugs and polypharmacy are associated with fall risk [36, 37]. Antidepressants can increase fall risk [36, 38] by contributing to the development of various symptoms, such as orthostatic hypotension, impaired attention, or movement disorders [38]. Antidepressant drug use was associated with reduced speed and reduced stride length in a previous study [3]. We re-ran our analyses excluding those individuals that took antidepressant medication (*N* = 8). This did not change our overall conclusions, in accordance with the findings of van Iersel et al. [6]. More detailed information on our sample’s medication is depicted in Online Resource 3.

A notable characteristic of our sample is a large difference in body height between the participant groups, which could not be explained by the slight difference in gender distribution only. DP were on average 13 cm smaller than HC. Previous studies showed that depressive symptoms were associated with smaller body height [39] and that larger body height results in larger speed and stride length [40]. Although correction for leg length did not change our overall conclusions, the difference in height—despite exact measurement—remains striking.

To our knowledge, this is the first study to examine both spatiotemporal gait parameters and MTC in depressed older patients with a high precision movement analysis system. Given that our results are largely in line with our expectations, replication in larger samples would be a promising endeavor to deepen our understanding of fall risk in psychiatric disease. It should be mentioned that according to our calculations in G*Power version 3.1.9.7 [41], we would have needed at least 28 persons in total to detect large within-subject effects and at least 74 persons in total to detect large between-subject effects (with *α* = 0.0025, power = 0.8, correlation among repeated measures = 0.5, partial eta-squared ≥ 0.14). Data collection was largely hampered by the Corona pandemic, however, as our target group belonged to the most vulnerable at risk group for the disease. We, thus, ended up with 35 persons in total. Therefore, the study might have been underpowered for the between-subject effects and to detected small to medium effects in general. A post hoc power sensitivity analysis revealed that with *α* = 0.0025, power = 0.8, total sample size = 35 and correlation among repeated measures = 0.5, effect sizes of partial eta-squared ≥ 0.11 should be detectable for within-subject effects, and partial eta-squared ≥ 0.27 for between-subject effects. Future studies should pay special attention to recruit larger well-mixed, representative samples and to assess medication in detail. It would be interesting to investigate whether gait deviations with initially depressive symptomatology might predict—and thus be a sign of—a prodromal phase of (neurological) diseases such as Parkinson’s. A major aim of future studies should be the development of targeted interventions to prevent falls—possibly by increasing muscle strength, balance, and fitness, by increasing step length (and, thus, MTC), by treadmill training toward more rhythmic stepping, teaching sensitive attention allocation, and/or by educating patients about their potentially higher fall risk due to the disease or medication. Overall, the maintenance of physical and cognitive functionality in old age should be treated with high priority in the context of interdisciplinary rehabilitation.

## Supplementary Information

Below is the link to the electronic supplementary material.

Supplementary file1 (PDF 581 KB)

Supplementary file2 (PDF 519 KB)

Supplementary file3 (PDF 501 KB)

Supplementary file4 (PDF 496 KB)

Supplementary file5 (PDF 555 KB)

## Data Availability

The datasets generated during and/or analyzed during the current study are available from the corresponding author on reasonable request.
