# Supplementary material for: Variability and symmetry of gait kinematics under dual-task performance of older patients with depression
Source: Aging Clin Exp Res. 2022 Nov 18;35(2):283–91. doi: 10.1007/s40520-022-02295-6 (PMC9895023; doi:10.1007/s40520-022-02295-6)
Supplement: Supplementary file 1 — Supplementary file1 (PDF 581 KB) [file 40520_2022_2295_MOESM1_ESM.pdf]

Online Resource 1 - Aging Clinical and Experimental Research

"Variability and symmetry of gait kinematics under dual-task performance of older patients with depression"

AUTHORS: Pia Jungen (MSc, pijungen@ukaachen.de)[1], João P. Batista (PhD, Postdoctoral Fellow, joao.batista@srh.de)[2,5], Miriam Kirchner (MD, M.Kirchner@alexianer.de)[3], Ute Habel (PhD, Full Professor, uhabel@ukaachen.de)[1,4], L. Cornelius Bollheimer (MD, Full Professor, cbollheimer@ukaachen.de)[2], Charlotte Huppertz (PhD, Postdoctoral Fellow, chhuppertz@ukaachen.de)[1]

AFFILIATIONS:

[1] Department of Psychiatry, Psychotherapy and Psychosomatics, Faculty of Medicine, RWTH Aachen University, Pauwelsstraße 30, 52074 Aachen, Germany

[2] Department of Geriatrics, Faculty of Medicine, RWTH Aachen University, Morillenhang 27, 52074 Aachen, Germany

[3] Alexianer Aachen GmbH, Alexianergraben 33, 52062 Aachen, Germany

[4] Institute of Neuroscience and Medicine 10, Research Centre Jülich, Wilhelm-Johnen-Straße, 52428 Jülich, Germany

[5] School of Physical Therapy, Campus Rheinland, SRH University of Applied Sciences, 51377 Leverkusen, Germany

CORRESPONDENCE:

Ms. Pia Jungen, Department of Psychiatry, Psychotherapy and Psychosomatics, Faculty of Medicine, RWTH Aachen University, Pauwelsstraße 30, 52074 Aachen, Germany. E-mail: pijungen@ukaachen.de; ORCID ID: 0000-0003-3206-2794; phone: +49/(0)241 80 37675

Online Resource 1. Results of mixed design ANOVAs for mean values of gait parameters, standardized based on leg length.[a]

	Speed mean				Stride length mean				Swing time mean				Minimum toe clearance mean			
	**df**	**F**	**p**	**η_p^2**	**df**	**F**	**p**	**η_p^2**	**df**	**F**	**p**	**η_p^2**	**df**	**F**	**p**	**η_p^2**
**Between-subject effects**																
**Group**	1	16.33	0.000*	0.33	1	21.61	0.000*	0.40	1	2.42	0.129	0.07	1	5.45	0.026	0.14
**Error (Group)**	33				33				33				33			
**Within-subject effects**																
**Condition**	1	29.55	0.000*	0.47	1	23.36	0.000*	0.41	1	13.27	0.001*	0.29	1	7.75	0.009	0.19
**Condition*Group**	1	1.49	0.230	0.04	1	1.87	0.181	0.05	1	1.73	0.198	0.05	1	1.07	0.310	0.03
**Error (Condition)**	33				33				33				33			

[a]*ANOVA = analysis of variance, df = degrees of freedom, F = F value, p p value, η_p^2 = partial eta-squared; *statistical significance with α<0.0025.*
