# Supplementary material for: Variability and symmetry of gait kinematics under dual-task performance of older patients with depression
Source: Aging Clin Exp Res. 2022 Nov 18;35(2):283–91. doi: 10.1007/s40520-022-02295-6 (PMC9895023; doi:10.1007/s40520-022-02295-6)
Supplement: Supplementary file 3 — Supplementary file3 (PDF 501 KB) [file 40520_2022_2295_MOESM3_ESM.pdf]

Online Resource 2b - Aging Clinical and Experimental Research

"Variability and symmetry of gait kinematics under dual-task performance of older patients with depression"

AUTHORS: Pia Jungen (MSc, pijungen@ukaachen.de)[1], João P. Batista (PhD, Postdoctoral Fellow, joao.batista@srh.de)[2,5], Miriam Kirchner (MD, M.Kirchner@alexianer.de)[3], Ute Habel (PhD, Full Professor, uhabel@ukaachen.de)[1,4], L. Cornelius Bollheimer (MD, Full Professor, cbollheimer@ukaachen.de)[2], Charlotte Huppertz (PhD, Postdoctoral Fellow, chhuppertz@ukaachen.de)[1]

AFFILIATIONS:

[1] Department of Psychiatry, Psychotherapy and Psychosomatics, Faculty of Medicine, RWTH Aachen University, Pauwelsstraße 30, 52074 Aachen, Germany

[2] Department of Geriatrics, Faculty of Medicine, RWTH Aachen University, Morillenhang 27, 52074 Aachen, Germany

[3] Alexianer Aachen GmbH, Alexianergraben 33, 52062 Aachen, Germany

[4] Institute of Neuroscience and Medicine 10, Research Centre Jülich, Wilhelm-Johnen-Straße, 52428 Jülich, Germany

[5] School of Physical Therapy, Campus Rheinland, SRH University of Applied Sciences, 51377 Leverkusen, Germany

CORRESPONDENCE:

Ms. Pia Jungen, Department of Psychiatry, Psychotherapy and Psychosomatics, Faculty of Medicine, RWTH Aachen University, Pauwelsstraße 30, 52074 Aachen, Germany. E-mail: pijungen@ukaachen.de; ORCID ID: 0000-0003-3206-2794; phone: +49/(0)241 80 37675

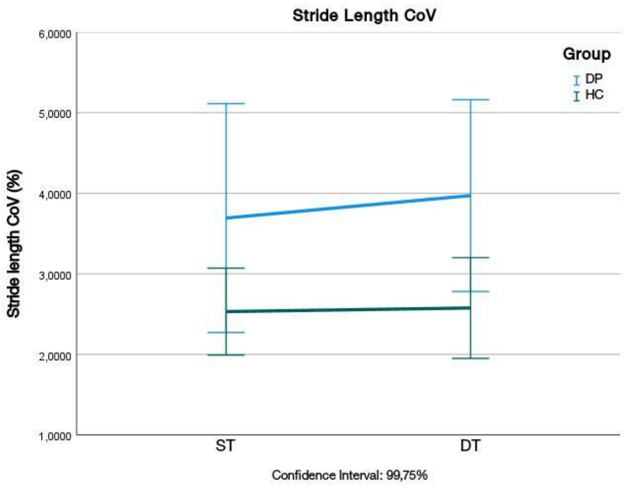

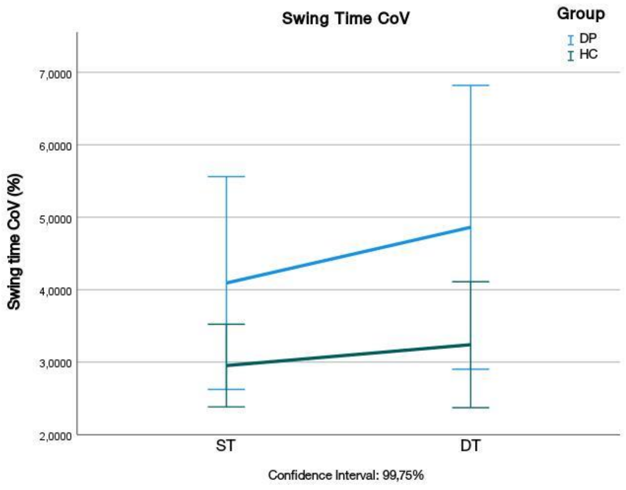

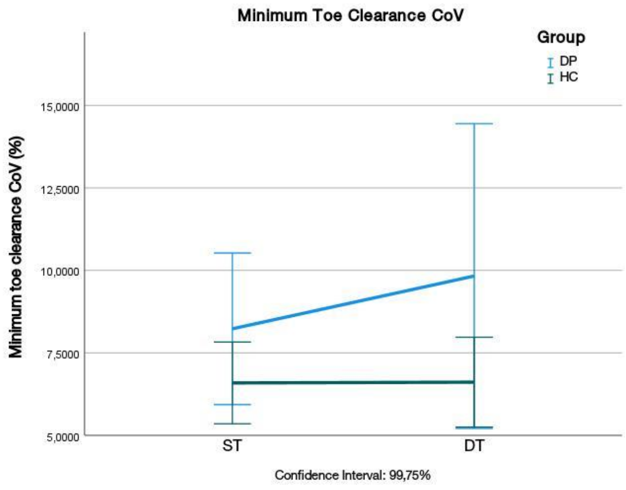

Online Resource 2b. Graphs of coefficients of variation (CoV) of gait parameters for depressed patients (DP) and healthy controls (HC) under single-task (ST) and dual-task (DT) performance.
